# Schizophrenia around the time of pregnancy: leveraging population-based health data and electronic health record data to fill knowledge gaps

**DOI:** 10.1192/bjo.2020.78

**Published:** 2020-08-28

**Authors:** Clare L. Taylor, Trine Munk-Olsen, Louise M. Howard, Simone N. Vigod

**Affiliations:** Women's College Hospital, Canada; Department of Economics and Business Economics, Aarhus University, Denmark; Women's Mental Health, Institute of Psychiatry, Psychology and Neuroscience, Kings College London, UK; Women's College Research Institute, Women's College Hospital, Canada

**Keywords:** Schizophrenia, pregnancy, information technologies, epidemiology, perinatal psychiatry

## Abstract

**Background:**

Research in schizophrenia and pregnancy has traditionally been conducted in small samples. More recently, secondary analysis of routine healthcare data has facilitated access to data on large numbers of women with schizophrenia.

**Aims:**

To discuss four scientific advances using data from Canada, Denmark and the UK from population-level health registers and clinical data sources.

**Method:**

Narrative review of research from these three countries to illustrate key advances in the area of schizophrenia and pregnancy.

**Results:**

Health administrative and clinical data from electronic medical records have been used to identify population-level and clinical cohorts of women with schizophrenia, and follow them longitudinally along with their children. These data have demonstrated that fertility rates in women with schizophrenia have increased over time and have enabled documentation of the course of illness in relation with pregnancy, showing the early postpartum as the time of highest risk. As a result of large sample sizes, we have been able to understand the prevalence of and risk factors for rare outcomes that would be difficult to study in clinical research. Advanced pharmaco-epidemiological methods have been used to address confounding in studies of antipsychotic medications in pregnancy, to provide data about the benefits and risks of treatment for women and their care providers.

**Conclusions:**

Use of these data has advanced the field of research in schizophrenia and pregnancy. Future developments in use of electronic health records include access to richer data sources and use of modern technical advances such as machine learning and supporting team science.

## Background

Schizophrenia is a severe mental disorder characterised by distortions in thinking, language, perception, emotions and sense of self. It often includes psychotic experiences, such as hearing voices or delusions and can be chronic and disabling for many with the condition.^1^ Prevalence is usually estimated at between 0.5 and 1%^2^ although the Global Burden of Disease study 2016 gave a global age-standardised point prevalence of 0.28%.^3^ Schizophrenia is ranked 12 among 310 diseases and injuries causing disability.^4^ Age at onset is typically in late adolescence to early adulthood, but in women is typically about 5 years later than in men, at the height of a woman's reproductive years.^5^

## Introduction to the evidence base in the field of schizophrenia and pregnancy

Most published research in the area of schizophrenia and pregnancy derives from case studies, qualitative work and small cohort studies.^6–13^ Small studies have shown that with practical and emotional supports, many women with schizophrenia are able to parent and be confident and successful as mothers.^14^ However, this research has also identified some specific needs among women with schizophrenia in relation to pregnancy, including in prenatal care, postpartum monitoring for early detection of psychiatric symptoms, and support with breastfeeding and parenting.^15^ These small studies have shown that mothers with schizophrenia are at disproportionately high risk for being single parents,^16^ social care involvement^14,17^ and custody loss.^14^ Further, there have been rare reports of pregnant women with schizophrenia having delusions around the fetus and denial of pregnancy^18,19^ and in rare but tragic circumstances, suicide and infanticide.^20–22^

The evidence, based mainly on case reports and small observational studies, has been important for raising awareness. However, small clinical studies are somewhat limited in their ability to generate data that fully characterises women and children in this potentially vulnerable maternal–child population. These types of study designs do not generally have the sample size or representativeness to allow for prediction of which women with schizophrenia will be at risk for which outcomes, and do not support accurate estimation of true incidence and prevalence of outcomes in the population, or whether, how and in whom some of the negative outcomes could be prevented.^12^ Small studies are also underpowered to provide accurate information on the risk for rare but important medical and mental health outcomes for mothers and their children, and the benefits and risks of antipsychotic medication – a mainstay of treatment in schizophrenia – in pregnancy.^23^ However, recruiting enough pregnant and postpartum women with schizophrenia to conduct high-quality clinical studies with large, representative samples results is time consuming and costly.

Secondary analysis of routinely collected healthcare data for research is an attractive strategy to create generalisable information to guide public policy and patient care in this area. Routinely collected healthcare data can allow research studies to be rapidly and efficiently conducted by accessing data about large numbers of women with and without schizophrenia, over long periods of time, with linked data on important risk factors and outcomes. In recent years, increased digitisation of clinical records has enabled increasing amounts of detailed routinely collected health data to be leveraged for use in medical research. Herein, we present examples of research conducted by our research teams using three routinely collected healthcare data sources in Canada, Denmark and the UK, to illustrate key advances that have been made in the area of schizophrenia and pregnancy by creating and analysing representative cohorts of women with schizophrenia around the time of pregnancy. We also present our hopes for how to advance this work further in the future.

## Method

We undertook a narrative review of research from our own research teams in Canada, Denmark and the UK using routinely collected health data to illustrate key advances in the area of schizophrenia and pregnancy.

### Data sources

Population-level health registers and clinical electronic health records represent two main types of routinely collected healthcare data that we have leveraged for analyses in populations of women with schizophrenia around the time of pregnancy.

Population-level health registers – sometimes called healthcare administrative data – contain structured data including standard demographic information (for example dates of birth and death, sex, region of residence), dates of out-patient, emergency department and in-patient healthcare contacts, and medical diagnoses and procedures. In these registers, an individual's healthcare data can be de-identified and/or anonymised and linked across data-sets, accessed and analysed quantitatively. These data sources cover entire populations and have a high degree of external validity, ideal for public health research, surveillance and policymaking.^24^ They are regulated by government bodies, with strict privacy requirements to protect the integrity of the data, and to avoid the risk for re-identification of an individual from research using the records. For example, in all the data-sets with which we work, it is prohibited to report on cell sizes smaller than 5–10 people, to protect anonymity.

In Ontario, Canada's largest province (population ~14.6 million), multiple sources of administrative health records have been linked to each other using a unique patient identifier common across all health records, dating back to the late 1980s. These data-sets, housed at ICES (formerly the ‘Institute for Clinical Evaluative Sciences’) – the designated Provincial repository for the secure collection and analysis of healthcare data for research – include hospital admissions, emergency department visits, medication and immigration records (www.ices.on.ca). Maternal and infant records are linkable to each other via hospital delivery records. In Denmark, a unique personal identification number ensures linkage within and between a range of population registers, with data including information on hospital admissions, births, spontaneous and induced abortions, fertility treatment, social care, education and also linkage across family members including fathers’ and children's’ health dating back in many cases to the mid-1960s (www.cirrau.au.dk/data-resources).

Most health administrative data-sets do not contain detailed information about clinical signs and symptoms. This is changing, with novel linkages of population-level clinical registers, where clinicians are mandated to enter clinical data such as symptoms and medical assessments for entire populations into population-level administrative health data registers. For example, in Ontario, there is a linked population-level mental health hospitalisation data-set, the Ontario Mental Health Reporting System, where clinical staff enter detailed data, which includes symptom-level data, for every patient through admission and discharge.^25^ More recently, a clinical maternity registry, BORN Ontario (Better Outcomes Registry and Network), has also been linked (www.bornontario.ca). This comprises clinical records sourced from maternity and neonatal services across the province, which provide more detailed data including sociodemographic data at the individual level (for example marital and education status), behavioural risk factor information (for example body mass index, smoking, intimate partner violence), some information on fathers, and details on medical testing, such as blood results, which are lacking in administrative data.

In the UK, advances in technology have enabled access to the routinely collected data from clinical electronic health records with in-depth information, including risk assessments, family histories, safeguarding assessments and that include clinical notes written as free text. These can be de-identified for use in research, by transforming free text into structured coded data for quantitative analysis. The South London and Maudsley (SLAM) NIHR Biomedical Research Centre (SLAM BRC) Clinical Record Interactive Search (CRIS) system is a source of clinical secondary mental healthcare data covering four London boroughs that includes all secondary mental healthcare (i.e. psychiatry) service use for that population.^26,27^ CRIS was set up in 2007 to allow researchers to access full but pseudo-anonymised data from the SLAM clinical records. CRIS hosts a de-identified data resource allowing searching and retrieval functionality for information from SLAM patient records including text fields (for example events notes, correspondence) so that bespoke research databases can be assembled. Fully electronic (i.e. paperless) health records have been maintained across all SLAM services since April 2006. CRIS de-identification performance and security protocols have been published^28^ and it has been approved as a source of secondary data for research since 2008.

CRIS has also been linked to other sources of data including local primary care data, national pupil data, mortality records and UK hospital data by the South London and Maudsley Clinical Data Linkage Service, transferring encrypted information via a secure firewall.^26^ Using patient data in this research does not require women to consent. Patients are given the opportunity to ‘opt out’ if they do not wish their information to be included in research. Through various ongoing dissemination activities, patients and public are informed about ongoing research and are also invited to give their input on research plans.

## Results

We identified four research themes from work conducted by our teams where population health registers and electronic clinical records have contributed to the evidence base and enabled us to make scientific advances in our understanding of women with schizophrenia and their reproductive health. These are:

- generalisable samples;
- longitudinal follow-up;
- rare outcomes;
- comparator groups.

For each we present the generated evidence and highlight strengths and challenges of using these data sources in this line of research.

### Generalisable samples

The volume of data in electronic health records and population registries and their coverage of populations has enabled access to representative samples of women with schizophrenia. However, data such as physician- and hospital-based diagnoses are not collected for research purposes, and can be subject to data entry errors. To generate cohorts of women with schizophrenia who have a pregnancy, utilising these data-sets has required substantial data cleaning and development of algorithms to define and validate health indicators.

In Ontario, studies have included all women in the province of Ontario^29^ and researchers have developed and validated an algorithm to identify individuals with schizophrenia in health administrative data^30^ using data from two sources – hospital admissions data and out-patient data. Researchers used ICD diagnostic codes for schizophrenia and schizoaffective disorder arising from a hospital admission or three out-patient visits within 3 years. This definition has a sensitivity of 90.1, specificity of 68.0; a positive predictive value of 77.1 and a negative predictive value of 88.7 compared to a clinical record.

Research from Denmark has included all women with past admissions for psychiatric disorder across the whole country. Studies have used ICD diagnoses from the Danish Psychiatric Central Research Register to identify schizophrenia.^31^ Early data used hospital admissions only but since 1995 the register has contained both hospital admissions and out-patient data. Validation of ICD-10 first time diagnoses of schizophrenia in the register of in-patients and out-patients against clinical case notes has been reported as having a positive predictive value of 89.7–97.5%.^32^

In the UK, research in women with schizophrenia and pregnancy initially used routinely collected primary care data, which gave rise to fairly small samples.^33,34^ The secondary mental health data from CRIS has allowed research to be carried out on representative samples of women managed in secondary care, but will miss those few women managed solely in primary care.^35^ In CRIS, natural language processing applications have been developed for text mining, using General Architecture for Text Engineering^36^ to derive structured data from free-text fields, taking into account the textual context of words/phrases of interest (for example distinguishing between positive and negative statements or past and present occurrences). These applications are developed using an iterative process and validated against ‘gold standard’ manual annotations of documents by calculating precision (positive predictive value) and recall (sensitivity).^36^ Research focused on pregnant women with a history of severe mental illness has used an application developed to extract diagnoses of severe mental illness prior to pregnancy, reading free-text notes to resolve any ambiguities.^35,37,38^

Thus, schizophrenia can be validly and reliably identified in routinely collected or ‘real-world’ health administrative and clinical data, opening up multiple opportunities for our own teams and for researchers around the world to rapidly study this population using these or similar data sources.

### Longitudinal follow-up

As a result of the rapid accumulation of large volumes of data occurring over long periods of time, secondary analysis of healthcare data allows for efficient measurement of trends over time, as well as long-term follow-up of mothers and their children. An important application of this in the area of schizophrenia in pregnancy is research on time trends in fertility rates, as well as characterisation of the epidemiology of the course of psychiatric illness in relation with pregnancy.

Using population registry data, birth rates among women with schizophrenia have been measured by capturing records of births recorded in hospital data^39^ or civil registration data.^40^ Early research from the UK using primary care data showed lower birth rates in women with psychotic disorders than who were matched population controls.^34^ Research from Denmark showed that birth rates were lower in women with schizophrenia, than the general population and women with other mental disorders even when accounting for higher rates of terminations in this group of women.^40^ Research using the ICES data-sets in Ontario, Canada examined trends in birth rates in women with schizophrenia over time and found increases of 16% from 1996–1998 to 2007–2009.^39^ By the end of the study period, birth rates were similar between women with and without schizophrenia in the under 25 age group. These findings have shown that over time, more women with schizophrenia are becoming mothers, and have supported the argument for urgent research to determine how best to support this vulnerable group in their health and the mental and physical health of their children.^41^

Our research teams have also focused on gaining a better understanding of the course of a woman's psychiatric illness across pregnancy and postpartum. Course of illness in these large data-sets has generally been measured by identifying psychiatric hospital admissions to determine severe psychiatric relapses. Data from Canada has reported admissions during pregnancy at 12–23%^29,42^ in women with a history of schizophrenia and data from the UK has reported admissions during pregnancy at 24% in women with a history of non-affective psychosis who were in contact with secondary mental healthcare.

In the first 3 months postpartum, UK data reported 31% of women with non-affective psychosis were admitted.^38^ The figures reported for the first year postpartum were 19% and 16% in data from Canada and Denmark, respectively,^42,43^ possibly reflecting the different population in the UK – a secondary care cohort – whereas those for Denmark and Canada are from an epidemiologically representative primary and secondary care population cohort. Although there is some variability across data-sets, these data have consistently documented that women with schizophrenia are at high risk of admission in pregnancy and postpartum and that early postpartum is the highest risk for admission across the whole perinatal period.^42,43^

In Canada, Denmark and the UK, our teams have also shown that markers of the severity of the psychiatric disorder such as number and recency of previous admissions are predictive of admissions in the perinatal period.^37,43,44^ Interestingly, in a Canadian study, having a consistent mental healthcare provider in pregnancy (either a primary care provider or a psychiatrist) was associated with a lower likelihood of requiring psychiatric admission in the postpartum. Other predictors of postpartum psychiatric admission were age >34, low income, not having an ultrasound before 20 weeks’ gestation as well as psychiatric comorbidity and prior psychiatric hospitalizations.^44^

Conducting longitudinal research relying on clinical studies prospectively collecting data across years would be very costly and at high risk of substantial loss to follow-up across the course of pregnancy and postpartum. These resources of prospective routinely collected healthcare data include more women with longer follow-up periods, minimising attrition, and can include otherwise hard to reach women too unwell to take part in a clinical study. Measuring psychiatric outcomes using hospital admission data may not be as sensitive an indicator of a woman's mental health as a clinical assessment. However, these findings inform clinicians that women with schizophrenia are at risk of a severe relapse in the perinatal period and require close monitoring during this period to prevent relapse, particularly in the early postpartum where admission might involve separating mother and baby, as well as determining which women may be at particular risk and hence require extra support.

Carrying out life-course research, including following women for their reproductive trajectories, and intergenerational research (i.e. research on outcomes of mothers and outcomes of their children) in healthy populations by recruiting mothers and babies has proved increasingly difficult. In the UK, a new large cohort study (the LIFE study) could not recruit to target and was stopped (www.lifestudy.ac.uk). Conducting clinical life-course and intergenerational research for women with schizophrenia would be an even greater challenge. More recently, routinely collected healthcare data has increasingly integrated data from across generations, different phases of life and critical periods of development, and research has begun to address longer-term follow-up of both women and children. For example, Canadian data have shown that women with schizophrenia are at higher risk of repeat pregnancy within 12 months of an index live birth than women without schizophrenia.^45^ Danish data through linkage to social care data and other family members, has also shown high risk of out-of-home placement in children of women with severe mental disorders^46,47^ and long-term follow-up of mental health records have shown children of parents with severe mental disorders have higher incidence of child and adolescent mental disorders up to age 17 compared with children of parents without severe mental disorders.^48^

### Rare outcomes

Many obstetric and neonatal complications are relatively uncommon in the general population. For example, the rate of stillbirths in 2015 was 18.4 per 1000 live births globally and 3.4 in high-income countries/developed regions^49^ and in 2017, globally, neonatal death was reported at 18 per 1000 live births and 3 per 1000 live births in high-income countries.^50^ Pre-eclampsia has been estimated at 4.6%^51^ and rates of preterm birth have been reported at 8.7% in Europe and 11.2% in North America.^52^ Given that schizophrenia is already a rare disorder, detecting such outcomes in these women requires very large samples. Using population-level data and including a variety of sources of linked data, research from Canada and Denmark has shown that compared with women in the population who do not have a history of schizophrenia, women with schizophrenia tend to have higher risk outcomes including pre-eclampsia, venous thromboembolism, placental complications and haemorrhage,^29^ assisted deliveries such as caesarean sections, inductions of labour and maternal intensive care admissions.^29,53^

Regarding neonates; data from Canada have also found increased risk for preterm birth, small for gestational age, large for gestational age and low birth weight and an increased risk for other neonatal morbidities such as seizures, sepsis, and neonatal abstinence syndrome,^29^ and from Denmark; poorer Apgar scores.^53^ Earlier research from the UK using primary care data has shown that women with history of psychotic disorder, compared with matched controls had more caesarean sections and higher proportion of stillbirths and neonatal deaths.^33^

Recent research from Canada using clinical birth registry data (BORN) to look at mediators of elevated risk, found that smoking in women with schizophrenia explained the greatest proportion of elevated risks for preterm, small-for-gestational-age scores and Apgar scores. Other relevant mediators were illicit substance use and reproductive history including prior preterm birth, caesarean section, surgical abortion and assisted reproduction.^54^

Regarding rarer psychiatric outcomes, research in Canada and the UK has looked at self-harm in pregnancy, detecting rates of 1% using emergency department visits^42^ and 11.4% in women with non-affective psychosis in secondary mental healthcare using clinical notes.^55^ Research using the Danish population (1 202 294 women) identified 1554 women with a severe first-onset postpartum mental disorder, of whom 64 had a hospital record of self-harm following diagnosis. Of these, 6 (9.4%) women had a diagnosis of schizophrenia or related disorder.^56^ Canadian research has also looked at suicide among women over a 15-year period in the province of Ontario identified using death records. Of 1914 suicides identified, 51 occurred during the perinatal period, but among women with psychotic disorders in the previous 5 years, numbers were too small to report,^57^ indicating that suicide among women with schizophrenia in the perinatal period is very rare.

Challenges with using routinely collected healthcare data to identify rare outcomes include the risk of false-positive findings when making multiple comparisons, statistical differences that may not be clinically meaningful and residual confounding. Important covariates such as partner abuse or childhood maltreatment are not usually recorded in administrative data, but some of these are potentially extractable from comprehensive electronic health records such as CRIS, and with linkage of clinical registry data, such as with BORN in Ontario. Despite these limitations, these data allow us to determine what is happening on a ‘real-world’ level and no other method of research would provide sufficient data to detect these outcomes with population-level coverage and with such a high degree of external validity. These data give us information at the public health level regarding adverse health outcomes for mother and baby in women with schizophrenia enabling us to develop targeted interventions to address identified health disparities in this group of women and their babies.

### Comparator groups

Another and often overlooked advantage of using population-based data is they have allowed for novel and creative ways of identifying multiple and varying control or comparison groups. Schizophrenia is primarily managed with antipsychotic medication and one of the biggest clinical concerns for patients and healthcare providers is how to weigh the risks and benefits of medication use in pregnancy. Applying modern statistical methods, such as propensity score matching, to the question of the safety of antipsychotic medication use in pregnancy has led to advances in knowledge about the risk–benefit ratio of the use of these medications in pregnancy.

For example, research from Canada has investigated maternal physical health and perinatal outcomes in women exposed and unexposed to antipsychotic medication in pregnancy.^58^ In an unmatched comparison, the antipsychotic-exposed women had higher risk of gestational diabetes and hypertensive disorders, and higher risks of adverse labour-, delivery- and neonatal-related outcomes. These differences disappeared when the antipsychotic-exposed group was compared to a propensity-matched unexposed group, indicating that factors other than medication may account for these differences.

Prescribing and exposure to psychotropic medication in pregnancy remains an area of contention for women and clinicians. The rich source of clinical psychiatric records and use of natural language processing applications to extract medication information in UK data from CRIS has enabled us to look in detail at changes in exposure status to medication in pregnancy in women with schizophrenia, including stops and switches in medications. Changes may reflect concerns by clinicians and women regarding effects of these medications during pregnancy, suggesting need for more evidence on the risks and benefits of medications in pregnancy. Around 40% of women with a history of non-affective psychosis stopped or switched a medication in the first trimester.^35^ We also determined the reasons for changes and whether stops in medication were a woman or a clinician's decision and whether medication was discontinued gradually or abruptly. Given there were many complex changes in medication throughout pregnancy in some women we were unable to report by pregnancy whether medication discontinuation was a woman or clinician's decision. However, when women stopped medications themselves, all stopped abruptly. Reasons for stopping or switching medications were primarily as a result of becoming pregnant. Other reasons included side-effects, improved symptoms or ‘non-compliance’.^35^ We have also investigated medication exposure as a predictor of psychiatric relapse in pregnancy and postpartum. Comparing within women with a history of psychotic disorders, these data have shown that women on no medication tended to have greater risk of relapse in pregnancy than women exposed to medication.^37^

In Denmark, researchers are also utilising new and creative methods for investigating psychotropic medication in pregnancy and have attempted to account for confounding by indication among antidepressant users for outcomes in offspring by comparing unexposed women, women exposed in pregnancy only, women exposed before pregnancy only, as well as before and during pregnancy. To account for any specific effects of intrauterine influence of medication use on child health, fathers were also used as negative controls.^59^ Note, this approach has not yet been carried out with respect to antipsychotic medications.

There are limitations to using routinely collected healthcare data for medication research. Adherence is largely unknown, given that much of the data is prescription related, leading to potential misclassification of medication exposures. Use of clinical psychiatric records in UK data have been able to give a clearer picture regarding whether women in secondary care are taking medication, although the quality of clinical notes varies, which may depend again on women's engagement with services and the severity of their psychiatric illness. However, in the absence of randomised controlled trial data, utilisation of population scale records and detailed clinical data, as well as newer analytic techniques are increasing the likely validity of these findings.

## Discussion

### Research in schizophrenia and pregnancy: future directions?

New methods are consistently being developed to enrich the variety of data sources that will give access to greater quality of information about pregnant women and new mothers with schizophrenia, and their children. The UK data also includes clinical outcome data, including from the Health of the Nation Outcome Scales,^60^ designed to assess level of functioning in people with mental illness. Adding patient reported outcome measures will also give us access to standardised measures across jurisdictions. A large new programme is underway in the UK called ELIXIR (Early Life data Cross Linkage in Research)^61^ using detailed local maternity data in South East London, facilitating linkage of data from mothers and babies and linkages with multiple sources of data from primary and secondary care, and local schools, enabling detailed child follow-up. In the Ontario data, additional linkage of social service, child welfare and child educational assessment data, will be linked to health data, which will enable future opportunities for research.

Further exciting opportunities are related with plans to increase the varieties of data linked with these large population and clinical data sources, such as imaging data, genetics data, bio-resources and social media. This will facilitate multidisciplinary research into biology, causes, mechanisms and medical treatments, as well as social determinants. Work is already underway in Denmark and the UK to incorporate biological data with these clinical and administrative data sources. In Denmark, a large-scale research effort iPSYCH (http://iPSYCH.au.dk/) was initiated in 2012 with an outlined purpose of studying genetic and environmental risk factors for mental disorders including schizophrenia and how these interact.^62^ The genetic data stems from a national biobank and the information is linked with a range of existing population registers allowing for longitudinal studies of individuals across decades.

As the volume and complexity of data grows, we will have more opportunity to employ newer methodologies of causal inference such as propensity score matching, instrumental variables and other epidemiological study designs such as self-controlled case series,^63,64^ and we can start to simulate experimental data, supporting evaluation of interventions and treatment programmes, and future research may also look at effectiveness and cost-effectiveness of health services such as mother and baby units, community services and social care. Further, use of data mining techniques and more recently, techniques involving machine learning, have facilitated analysis of unstructured data and natural language processing applications have now been developed to extract psychiatric symptomatology in the UK data,^65^ giving access to more detailed information on psychiatric illnesses and severity. Future uses of machine learning are bringing us closer to more efficient ways of processing and analysing huge volumes of complex data, taking us toward a ‘big data’ model and possibilities of using predictive modelling to assist clinical decision-making and personalised medicine.

### Implications

It is our view that team science across international boundaries provides potential to fill research gaps including researching other rare outcomes, different interventions and populations. This involves collaboration across investigators with diverse skills and experiences, leading to greater integration of knowledge and information than individual investigators can achieve working independently. Integrating data across countries can further increase the external validity cross-culturally and across different health service models.

Use of these routinely collected health data also requires knowledge and expertise in different areas including medicine and health services, medical sciences, information technology infrastructure and data analytics, particularly as access to increased variety of data sources is increasing. In line with this, we have already published a multicentre meta-analysis using data from six countries including Canada, Denmark and the UK, looking at exposure to lithium in pregnancy,^66^ finding increased risk of neonatal admissions within 28 days of birth and increased risk of major malformations but not cardiac malformation, which has been traditionally associated with lithium use in pregnancy. This study has documented both feasibility and relevance of pooling these data sources internationally.

In conclusion, producing generalisable research that advances knowledge about how to care for women with schizophrenia around the time of pregnancy has been a challenge because of the prevalence of the disorder itself and challenges in engaging representative samples in clinical research. Secondary analysis of routinely collected healthcare data has contributed to scientific advances in four main areas of research that would otherwise be virtually impossible– creation of cohorts and health indicators on a population level, longitudinal follow-up and analysis of rare outcomes and use of comparator groups to limit selection bias. Future directions may enable access to richer sources of data, support development of personalised medicine, assist with clinical decision-making and incorporate biological resources, as well as collaborate across countries and research disciplines, to ensure optimal outcomes for pregnant women and new mothers with schizophrenia, and the health and well-being of their children across the lifespan.

## Data Availability

No original data are reported in this manuscript.

## References

[1] World Health Organization. *Schizophrenia* WHO, 2018 (https://www.who.int/news-room/fact-sheets/detail/schizophrenia).

[2] Goldner EM, Hsu L, Waraich P, Somers JM. Prevalence and incidence studies of schizophrenic disorders: a systematic review of the literature. Can J Psychiatry 2002; 47: 833–43. 12500753 10.1177/070674370204700904

[3] Charlson FJ, Ferrari AJ, Santomauro DF, Diminic S, Stockings E, Scott JG, Global epidemiology and burden of schizophrenia: findings from the global burden of disease study 2016. Schizophr Bull 2018; 44: 1195–203. 29762765 10.1093/schbul/sby058 PMC6192504

[4] Vos T, Abajobir AA, Abate KH, Abbafati C, Abbas KM, Abd-Allah F, Global, regional, and national incidence, prevalence, and years lived with disability for 328 diseases and injuries for 195 countries, 1990–2016: a systematic analysis for the Global Burden of Disease Study 2016. Lancet 2017; 390 1211–59. 28919117 10.1016/S0140-6736(17)32154-2 PMC5605509

[5] Rabinowitz J, Levine SZ, Hafner H. A population based elaboration of the role of age of onset on the course of schizophrenia. Schizophr Res 2006; 88: 96–101. 16962742 10.1016/j.schres.2006.07.007

[6] Auerbach JG, Hans SL, Marcus J, Maeir S. Maternal psychotropic medication and neonatal behavior. Neurotoxicol Teratol 1992; 14: 399–406. 1488034 10.1016/0892-0362(92)90050-k

[7] Babu GN, Desai G, Tippeswamy H, Chandra PS. Birth weight and use of olanzapine in pregnancy: a prospective comparative study. J Clin Psychopharmacol 2010; 30: 331–2. 20473073 10.1097/JCP.0b013e3181db8734

[8] McNeil TF, Kaij L, Malmquist-Larsson A. Women with nonorganic psychosis: mental disturbance during pregnancy. Acta Psychiatr Scand 1984; 70: 127–39. 6485846 10.1111/j.1600-0447.1984.tb01190.x

[9] Newport DJ, Calamaras MR, DeVane CL, Donovan J, Beach AJ, Winn S, Atypical antipsychotic administration during late pregnancy: placental passage and obstetrical outcomes. Am J Psychiatry 2007; 164: 1214–20. 17671284 10.1176/appi.ajp.2007.06111886

[10] Nguyen TN, Faulkner D, Frayne JS, Allen S, Hauck YL, Rock D, Obstetric and neonatal outcomes of pregnant women with severe mental illness at a specialist antenatal clinic. Med J Aust 2012; 196: 26. 10.5694/mja11.11152 25369845

[11] Peng M, Gao K, Ding Y, Ou J, Calabrese JR, Wu R, Effects of prenatal exposure to atypical antipsychotics on postnatal development and growth of infants: a case-controlled, prospective study. Psychopharmacology 2013; 228: 577–84. 23559219 10.1007/s00213-013-3060-6

[12] Seeman MV. Suicide among women with schizophrenia spectrum disorders. J Psychiatr Pract 2009; 15: 235–42. 19461398 10.1097/01.pra.0000351885.60507.1c

[13] Seeman MV. Gendering psychosis: the illness of Zelda Fitzgerald. Med Humanit 2016; 42: 65–9. 26392268 10.1136/medhum-2015-010734

[14] Seeman MV. Intervention to prevent child custody loss in mothers with schizophrenia. Schizophr Res Treat 2011; 2012: 796763 Available from: 10.1155/2012/796763. PMC3420381 22966446

[15] Seeman MV, Gupta R. Selective review of age-related needs of women with schizophrenia. Clin Schizophr Relat Psychoses 2015; 9: 21–9. 23471090

[16] Ranning A, Munk Laursen T, Thorup A, Hjorthoj C, Nordentoft M. Children of parents with serious mental illness: with whom do they grow Up? A prospective, population-based study. J Am Acad Child Adolesc Psychiatry 2016; 55: 953–61. 27806863 10.1016/j.jaac.2016.07.776

[17] Howard L, Thornicroft G, Salmon M, Appleby L. Predictors of parenting outcome in women with psychotic disorders discharged from mother and baby units. Acta Psychiatr Scand 2004; 110: 347–55. 15458558 10.1111/j.1600-0447.2004.00375.x

[18] Ostler T, Kopels S. Schizophrenia and filicide. Curr Womens Health Revs 2010; 6: 58–62.

[19] Solari H. Psychotic denial of pregnancy. Curr Womens Health Revs 2010; 6: 22–7.

[20] Austin M, Kildea S, Sullivan E. Maternal mortality and psychiatric morbidity in the perinatal period: challenges and opportunities for prevention in the Australian setting. Med J Aust 2007; 186: 364. 17407434 10.5694/j.1326-5377.2007.tb00940.x

[21] Cantwell R, Clutton-Brock T, Cooper G, Dawson A, Drife J, Garrod D, Saving Mothers’ Lives: reviewing maternal deaths to make motherhood safer: 2006–2008. The Eighth Report of the Confidential Enquiries into Maternal Deaths in the United Kingdom. BJOG 2011; 118: 1–205. 10.1111/j.1471-0528.2010.02847.x 21356004

[22] Spinelli MG. Maternal infanticide associated with mental illness: prevention and the promise of saved lives. Am J Psychiatry 2004; 161: 1548–57. 15337641 10.1176/appi.ajp.161.9.1548

[23] Galbally M, Snellen M, Power J. Antipsychotic drugs in pregnancy: a review of their maternal and fetal effects. Ther Adv Drug Saf 2014; 5: 100–9. 25083265 10.1177/2042098614522682 PMC4110873

[24] Harron K, Dibben C, Boyd J, Hjern A, Azimaee M, Barreto ML, Challenges in administrative data linkage for research. Big Data Soc 2017; 4(2); doi: 10.1177/2053951717745678. PMC6187070 30381794

[25] Martin L, Hirdes J, Morris J, Montague P, Rabinowitz T, Fries B. Validating the Mental Health Assessment Protocols (MHAPs) in the Resident Assessment Instrument Mental Health (RAI-MH). J Psychiatr Ment Health Nurs 2009; 16: 646–53. 19689558 10.1111/j.1365-2850.2009.01429.x

[26] Perera G, Broadbent M, Callard F, Chang C-K, Downs J, Dutta R, Cohort profile of the South London and Maudsley NHS Foundation Trust Biomedical Research Centre (SLaM BRC) Case Register: current status and recent enhancement of an Electronic Mental Health Record-derived data resource. BMJ Open 2016; 6: e008721. 10.1136/bmjopen-2015-008721 PMC4785292 26932138

[27] Stewart R, Soremekun M, Perera G, Broadbent M, Callard F, Denis M, The South London and Maudsley NHS foundation trust biomedical research centre (SLAM BRC) case register: development and descriptive data. BMC Psychiatry 2009; 9: 51. 19674459 10.1186/1471-244X-9-51 PMC2736946

[28] Fernandes AC, Cloete D, Broadbent MT, Hayes RD, Chang CK, Jackson RG, Development and evaluation of a de-identification procedure for a case register sourced from mental health electronic records. BMC Med Inform Decis Mak 2013; 13: 71. 23842533 10.1186/1472-6947-13-71 PMC3751474

[29] Vigod S, Kurdyak P, Dennis C, Gruneir A, Newman A, Seeman M, Maternal and newborn outcomes among women with schizophrenia: a retrospective population-based cohort study. BJOG 2014; 121: 566–74. 24443970 10.1111/1471-0528.12567

[30] Kurdyak P, Lin E, Green D, Vigod S. Validation of a population-based algorithm to detect chronic psychotic illness. Can J Psychiatry 2015; 60: 362–8. 26454558 10.1177/070674371506000805 PMC4542516

[31] Mors O, Perto GP, Mortensen PB. The Danish Psychiatric Central Research Register. Scand J Public Health 2011; 39 (7 Suppl): 54–7. 21775352 10.1177/1403494810395825

[32] Uggerby P, Ostergaard SD, Roge R, Correll CU, Nielsen J. The validity of the schizophrenia diagnosis in the Danish Psychiatric Central Research Register is good. Danish Med J 2013; 60: A4578. 23461991

[33] Howard LM, Goss C, Leese M, Thornicroft G. Medical outcome of pregnancy in women with psychotic disorders and their infants in the first year after birth. Br J Psychiatry 2003; 182: 63–7. 12509320 10.1192/bjp.182.1.63

[34] Howard LM, Kumar C, Leese M, Thornicroft G. The general fertility rate in women with psychotic disorders. Am J Psychiatry 2002; 159: 991–7. 12042188 10.1176/appi.ajp.159.6.991

[35] Taylor CL, Stewart R, Ogden J, Broadbent M, Pasupathy D, Howard LM. The characteristics and health needs of pregnant women with schizophrenia compared with bipolar disorder and affective psychoses. BMC Psychiatry 2015; 15: 88. 25886140 10.1186/s12888-015-0451-8 PMC4406022

[36] Cunningham H, Tablan V, Roberts A, Bontcheva K. Getting more out of biomedical documents with GATE's full lifecycle open source text analytics. PLoS Comput Biol 2013; 9: e1002854. 23408875 10.1371/journal.pcbi.1002854 PMC3567135

[37] Taylor CL, Broadbent M, Khondoker M, Stewart R, Howard LM. Predictors of severe relapse in pregnant women with psychotic or bipolar disorders. J Psychiatr Res 2018; 104: 100-7. 30015264 10.1016/j.jpsychires.2018.06.019

[38] Taylor CL, Stewart RJ, Howard LM. Relapse in the first three months postpartum in women with history of serious mental illness. Schizophr Res 2019; 204: 46–54. 30089534 10.1016/j.schres.2018.07.037

[39] Vigod SN, Seeman MV, Ray JG, Anderson GM, Dennis CL, Grigoriadis S, Temporal trends in general and age-specific fertility rates among women with schizophrenia (1996–2009): a population-based study in Ontario, Canada. Schizophr Res 2012; 139: 169–75. 22658526 10.1016/j.schres.2012.05.010

[40] Laursen TM, Munk-Olsen T. Reproductive patterns in psychotic patients. Schizophr Res 2010; 121: 234–40. 20570491 10.1016/j.schres.2010.05.018

[41] Jones I, Chandra PS, Dazzan P, Howard LM. Bipolar disorder, affective psychosis, and schizophrenia in pregnancy and the post-partum period. Lancet 2014; 384: 1789–99. 25455249 10.1016/S0140-6736(14)61278-2

[42] Rochon-Terry G, Gruneir A, Seeman MV, Ray JG, Rochon P, Dennis C-L, Hospitalizations and emergency department visits for psychiatric illness during and after pregnancy among women with schizophrenia. J Clin Psychiatry 2016; 77: 541–7. 27035409 10.4088/JCP.14m09697

[43] Munk-Olsen T, Laursen TM, Mendelson T, Pedersen CB, Mors O, Mortensen PB. Risks and predictors of readmission for a mental disorder during the postpartum period. Arch Gen Psychiatry 2009; 66: 189–95. 19188541 10.1001/archgenpsychiatry.2008.528

[44] Vigod SN, Rochon-Terry G, Fung K, Gruneir A, Dennis CL, Grigoriadis S, Factors associated with postpartum psychiatric admission in a population-based cohort of women with schizophrenia. Acta Psychiatr Scand 2016; 134: 305–13. 27437875 10.1111/acps.12622

[45] Gupta R, Brown HK, Barker LC, Dennis C-L, Vigod SN. Rapid repeat pregnancy in women with schizophrenia. Schizophr Res 2019; 212: 86–91. 31420202 10.1016/j.schres.2019.08.007

[46] Vigod SN, Laursen TM, Ranning A, Nordentoft M, Munk-Olsen T. Out-of-home placement to age 18 years in children exposed to a postpartum mental disorder. Acta Psychiatr Scand 2018; 138: 35–43. 29667167 10.1111/acps.12890

[47] Ranning A, Munk Laursen T, Thorup A, Hjorthoj C, Nordentoft M. Serious mental illness and disrupted caregiving for children: a nationwide, register-based cohort study. J Clin Psychiatry 2015; 76: e1006–14. 26335086 10.4088/JCP.13m08931

[48] Thorup AAE, Laursen TM, Munk-Olsen T, Ranning A, Mortensen PB, Plessen KJ, Incidence of child and adolescent mental disorders in children aged 0–17 with familial high risk for severe mental illness - A Danish register study. Schizophr Res 2018; 197: 298–304. 29132814 10.1016/j.schres.2017.11.009

[49] Blencowe H, Cousens S, Jassir FB, Say L, Chou D, Mathers C, National, regional, and worldwide estimates of stillbirth rates in 2015, with trends from 2000: a systematic analysis. Lancet Global Health 2016; 4: e98–108. 26795602 10.1016/S2214-109X(15)00275-2

[50] Hug L, Alexander M, You D, Alkema L, for Child UI-aG. National, regional, and global levels and trends in neonatal mortality between 1990 and 2017, with scenario-based projections to 2030: a systematic analysis. Lancet Global Health 2019; 7: e710–20. 31097275 10.1016/S2214-109X(19)30163-9 PMC6527519

[51] Abalos E, Cuesta C, Grosso AL, Chou D, Say L. Global and regional estimates of preeclampsia and eclampsia: a systematic review. Eur J Obstet Gynecol Reprod Biol 2013; 170: 1–7. 23746796 10.1016/j.ejogrb.2013.05.005

[52] Chawanpaiboon S, Vogel JP, Moller A-B, Lumbiganon P, Petzold M, Hogan D, Global, regional, and national estimates of levels of preterm birth in 2014: a systematic review and modelling analysis. Lancet Global Health 2019; 7: e37–46. 30389451 10.1016/S2214-109X(18)30451-0 PMC6293055

[53] Bennedsen B, Mortensen PB, Olesen AV, Henriksen TB, Frydenberg M. Obstetric complications in women with schizophrenia. Schizophr Res 2001; 47: 167–75. 11278134 10.1016/s0920-9964(99)00234-0

[54] Vigod SN, Fung K, Amartey A, Bartsch E, Felemban R, Saunders N, Maternal schizophrenia and adverse birth outcomes: what mediates the risk? Soc Psychiatry Psychiatr Epidemiol 2020; 55: 561–70. 31811316 10.1007/s00127-019-01814-7

[55] Taylor CL, van Ravesteyn LM, Lambregtse van denBerg MP, Stewart RJ, Howard LM. The prevalence and correlates of self-harm in pregnant women with psychotic disorder and bipolar disorder. Arch Womens Ment Health 2016; 19: 909–15. 27173485 10.1007/s00737-016-0636-2 PMC5021774

[56] Johannsen BMW, Larsen JT, Laursen TM, Bergink V, Meltzer-Brody S, Munk-Olsen T. All-cause mortality in women with severe postpartum psychiatric disorders. Am J Psychiatry 2016; 173: 635–42. 26940804 10.1176/appi.ajp.2015.14121510 PMC5325688

[57] Grigoriadis S, Wilton AS, Kurdyak PA, Rhodes AE, VonderPorten EH, Levitt A, Perinatal suicide in Ontario, Canada: a 15-year population-based study. CMAJ 2017; 189: E1085–92. 28847780 10.1503/cmaj.170088 PMC5573543

[58] Vigod SN, Gomes T, Wilton AS, Taylor VH, Ray JG. Antipsychotic drug use in pregnancy: high dimensional, propensity matched, population based cohort study. BMJ 2015; 350: h2298. 25972273 10.1136/bmj.h2298 PMC4430156

[59] Liu X, Agerbo E, Ingstrup KG, Musliner K, Meltzer-Brody S, Bergink V, Antidepressant use during pregnancy and psychiatric disorders in offspring: Danish nationwide register based cohort study. BMJ 2017; 358: j3668. 28877907 10.1136/bmj.j3668 PMC5594425

[60] Wing J, Beevor A, Curtis R, Park S, Hadden S, Burns A. Health of the Nation Outcome Scales (HoNOS). Research and development. Br J Psychiatry 1998; 172: 11–8. 9534825 10.1192/bjp.172.1.11

[61] NIHR Guy's and St Thomas’ Biomedical Research Centre. *eLIXIR Study* NIHR Guy's and St Thomas’ Biomedical Research Centre, 2018 (http://www.guysandstthomasbrc.nihr.ac.uk/our-work/research-themes/women-and-childrens-health/elixir-study/).

[62] Pedersen CB, Bybjerg-Grauholm J, Pedersen MG, Grove J, Agerbo E, Baekvad-Hansen M, The iPSYCH2012 case-cohort sample: new directions for unravelling genetic and environmental architectures of severe mental disorders. Mol Psychiatry 2018; 23: 6–14. 28924187 10.1038/mp.2017.196 PMC5754466

[63] Brauer R, Smeeth L, Anaya-Izquierdo K, Timmis A, Denaxas SC, Farrington CP, Antipsychotic drugs and risks of myocardial infarction: a self-controlled case series study. Eur Heart J 2015; 36: 984–92. 25005706 10.1093/eurheartj/ehu263 PMC4404491

[64] Swanson SA, Hernandez-Diaz S, Palmsten K, Mogun H, Olfson M, Huybrechts KF. Methodological considerations in assessing the effectiveness of antidepressant medication continuation during pregnancy using administrative data. Pharmacoepidemiol Drug Saf 2015; 24: 934–42. 26045370 10.1002/pds.3798 PMC4822692

[65] Jackson RG, Patel R, Jayatilleke N, Kolliakou A, Ball M, Gorrell G, Natural language processing to extract symptoms of severe mental illness from clinical text: the Clinical Record Interactive Search Comprehensive Data Extraction (CRIS-CODE) project. BMJ Open 2017; 7: e012012. 10.1136/bmjopen-2016-012012 PMC5253558 28096249

[66] Munk-Olsen T, Liu X, Viktorin A, Brown HK, Di Florio A, D'Onofrio BM, Maternal and infant outcomes associated with lithium use in pregnancy: an international collaborative meta-analysis of six cohort studies. Lancet Psychiatry 2018; 5: 644–52. 29929874 10.1016/S2215-0366(18)30180-9 PMC6077091

